# Antidepressants Fluoxetine Mediates Endoplasmic Reticulum Stress and Autophagy of Non–Small Cell Lung Cancer Cells Through the ATF4-AKT-mTOR Signaling Pathway

**DOI:** 10.3389/fphar.2022.904701

**Published:** 2022-05-10

**Authors:** Shali Shao, Xibing Zhuang, Lin Zhang, Tiankui Qiao

**Affiliations:** ^1^ Center for Tumor Diagnosis and Therapy, Jinshan Hospital, Fudan University, Shanghai, China; ^2^ Department of Emergency and Critical Care Medicine, Jinshan Hospital, Fudan University, Shanghai, China

**Keywords:** fluoxetine, non-small cell lung cancer, ATF4-AKT-mTOR signaling pathway, endoplasmic reticulum stress, autophagy

## Abstract

Fluoxetine, one of the latest clinical antidepressants, is reported to have the anti-proliferative effect on cancer cells *via* immune-related pathways. However, the mechanism is still not known. This study mainly focused on the discovery of the molecular basis of the inhibitory effect of fluoxetine in lung cancer. The specific anti-proliferation effect and autophagy induced by fluoxetine on lung cancer cell were shown in CCK8 and immunofluorescence. The RNA sequence hinted that the endoplasmic reticulum (ER) stress-related protein and mTOR pathway were enriched after fluoxetine treatment. Western blot results revealed that the ER stress pathway was activated by fluoxetine, including PERK, ATF4, and CHOP, while the AKT/mTOR pathway was inhibited. In addition, the transfection of ATF4 siRNA further discovered that ER stress participated in the inhibition of AKT/mTOR pathway and the induction of anti-proliferation and autophagy in the fluoxetine-treated cells. More importantly, fluoxetine was demonstrated to play cytotoxic activity in cancer cells without affecting normal cells. Our results showed that fluoxetine triggered the ATF4-AKT-mTOR signaling pathway to induce cell cycle arrest and autophagy restraining cancer cells’ growth in lung cancer. This study found fluoxetine unaffected the proliferation of normal lung epithelial cells, providing safe clinical therapeutic strategies for lung cancer patients with depression.

## Introduction

Lung cancer is a highly prevalent cancer worldwide with severe lethality, accounting for 30% of cancer-related death in China (Chen et al., 2016). Cancer patients suffer a high probability of getting depression, whose incidence is approximately 15% (Mitchell et al., 2011). To make the situation worse, the prevalence of depression in lung cancer, ranging from 16 to 29% (Hopwood and Stephens, 2000; Walker et al., 2006), is larger than the average of other cancers. The status of cancer will influence the process of depression and be influenced by the depression in turn. Specifically, long-lasting suffering and unhelpful effectiveness of anticancer treatment will boost depression and eventually speed up patients’ death (Akechi et al., 2008). Meanwhile, the influence of depression is multifaceted. Depression in cancer will accelerate the dysfunctional activation of the hypothalamic-pituitary axis; oxidative stress and inflammation; and a weakened immunosurveillance (Bortolato et al., 2017). Thus, therapeutical approaches that can simultaneously treat cancer and depression are valuable in clinic.

Antidepressants are widely used to cure depression. In the recent years, the tricycle antidepressants (TCA) were replaced by the newer generation antidepressants, because of the serious side effects. Fluoxetine, a selective serotonin reuptake inhibitor (SSRI), is one of the latest clinical antidepressants (Ginsburg, 2008). Reports demonstrated that fluoxetine also had an inhibitory effect on cancer progression in ovarian cancer (Lee et al., 2010), colon cancer, and breast cancer (Stepulak et al., 2008). However, in lung cancer the role that fluoxetine plays in cell death is still ambiguous.

The endoplasmic reticulum (ER) is an organelle that involves protein folding, calcium storage, and biosynthesis (Janssens et al., 2014). ER has a robust homeostasis system, but various factors can still destroy the balance, such as radiotherapy and chemotherapy. Under the stressed condition, the accumulation of misfolded or unfolded protein exceeds the ER protein folding capacity threshold, which will trigger ER stress (Bettigole and Glimcher, 2015). The ER stress can activate the HSP70-type BiP/GRP78, which dissociates from luminal domains to activate three related sensors (Bertolotti et al., 2000). PKR-like ER kinase (PERK), inositol-requiring enzyme 1α (IRE1α), and activating transcription factor 6α (ATF6α) are the main intraluminal ER proteins, and the activation of these proteins can trigger Unfolded Protein Responses (UPR) to maintain the homeostasis of ER (Bettigole and Glimcher, 2015). The role of ER stress is dual, either triggering cell death or cell survival, depending on the stress conditions and cell types (Jang et al., 2015; Shen et al., 2015). When the activation of UPR fails to cope with unfolded proteins to remain ER stability, it will trigger cell apoptosis, and in different malignancies, it can also cause autophagy to re-use the organelles (Lin et al., 2019). Accumulating evidences suggested that both ER stress and UPR are related to pathological processes, like cancer and depression (Lebovitz et al., 2015).

Here, we explore the anti-tumor potential of fluoxetine in lung cancer cells *in vitro*. We find that fluoxetine exerts an inhibitory role on cancer cells growth by inducing cell cycle arrest and autophagy without influencing normal cells. In addition, the ER stress-related pathway is involved in the anticancer treatment of fluoxetine. These results provide a new strategy of fluoxetine in the treatment of lung cancer patients with depression.

## Methods and Materials

### Cell Culture and Reagents

Paroxetine, fluoxetine, 3-MA, CQ were purchased by Sigma-Aldrich (Shanghai, China). The human lung cancer cell lines (H460 and A549) and normal lung epithelial cell (BEAS-2B) were supplied by Cell Bank (Shanghai, China). The human hepatoma cell line (Huh7) and normal liver cell line (L02) were gifted by Prof. Duo-Jiao Wu (Zhongshan Hospital, Shanghai, China). Primary antibody including p21 (2947), p27 (3686), CDK2 (18048), p62 (5114), LC3 (12741), PERK (5683), BIP (3177), ATF4 (11815), CHOP (2895), p-AKT (4060), AKT (9272), p-mTOR (2974), mTOR (2972), p-p70S6K (9234), p70S6K (97596), β-actin (3700), and GAPDH (5174) were bought from Cell Signaling Technology (Danvers, MA, United States). The cells were cultured in complete RPMI-1640 medium (Sigma, Louis, Missouri, United States) with 10% fetal bovine serum (FBS) (Biological Industries, Israel) at 37°C under 5% CO_2_.

### Cell Counting Kit-8 Assay

The cells were seeded in the 96-well plates (5000 cells/well). After 24 h of incubation, the cells were treated with fluoxetine for 24 h. Then, add 10ul/well CCK8 solution (Do jindo, Laboratories, Kumamoto, Japan) after changing the medium to FBS-free RPMI-1640 in the dark room. The cells were incubated at 37°C for 1 h. The OD value was gathered at 450 nm.

### Analysis of Cell Cycle

The cells were seeded in the 6-well plates (20 × 10^4^ cells/well). After 24 h of incubation, the cells were treated with the corresponding concentration of a drug. After 24 h, the cells were trypsinized and washed with PBS, and then, the cells were suspended with 70% ethyl alcohol at −20°C. The next day, the cells were stained with PI/RNase Staining Buffer (BD Bioscience) for 15 min after discarding the ethyl alcohol. Flow cytometer (Beckman Coulter) and ModFit LT 5.0 software were used to collect and analyze the results, respectively.

### Analysis of Cell Apoptosis

The cells were seeded in the 6-well plates (20 × 10^4^ cells/well). After 24 h of incubation, the cells were treated with the corresponding concentration of a drug. After 24 h, the cells were digested with EDTA-free trypsin and washed with Binding Buffer, and then, the cells were incubated with PI-FITC antibody (BD Biosciences) following the manufacturer’s instructions for 15 min. Flow cytometer (Beckman Coulter) was used to collect and analyze the results.

### Immunofluorescence

The cells were seeded in the 96-well plates (5000 cells/well). After 24 h of incubation, the cells were treated with the corresponding concentration of the drug for the next 24 h. After discarding the solution, the cells were fixed with 4% paraformaldehyde, then, the cells were permeabilized with 0.1% Triton X-100 and blocked with 5% BSA. The cells were incubated with LC3 antibody at 4°C overnight. On the next day, the cells were incubated with fluorophore-labeled secondary antibody for 1 h and washed with PBST, DAPI was used to stain the nucleus before observation. All of these steps were produced under dark condition.

### RNA-Sequencing Experiment

The cells were seeded in the 6-well plates (20 × 10^4^ cells/well). After 24 h of incubation, the cells were treated with the corresponding concentration of the drug for the next 24 h. After trypsining and washing, the total RNA was extracted from cells using TRIzol (Invitrogen). Library construction and sequencing were performed by HaploX Genomics Center (Jiangxi, China). The Cluster Analysis was processed with edge R (version 3.20.9) following manufacturer’s instructions. The libraries were sequenced on an Illumina PE150 platform and paired-end reads were generated. The Volcano Plot was achieved by DESeq2 (version 1.18.1). The analysis of Kyoto Encyclopedia of Genes and Genomes (KEGG) pathway was performed by the cluster Profiler (version 4.0.1).

### Small Interfering RNA Experiment

The siRNA sequences against ATF4 were synthesized by Gima Company: ATF4-siRNA, 5′-TCC​CTC​AGT​GCA​TAA​AGG​A-3′. NC-siRNA, 5′- UUCUCCGAACGUGUCACG A-3′. Transfecting cells with 5ul siRNA plasmid using 8 ul X-tremeGENE, based on the manufacturer’s instruction, and treating cells with the mentioned drugs after transfection.

### Western Blot

The cells were extracted with a mixture of SDS and PMSF. The protein samples were measured with a BCA assay kit (ThermoFisher, United States). Then, SDS-PAGE gel was used to distinguish the protein, PVDF membrane to transfer protein, and 5% milk to block protein. After that, the membrane was trimmed according to the marker and incubated with corresponding primary antibodies at 4°C overnight. On the next day, the membrane was washed with TBST and incubated with a secondary antibody. ECL (Millipore, Billerica, MA) was applied to detect the band which was needed.

### Statistical Analysis

Statistical differences between the compared groups were determined by one-way ANOVA analysis and Tukey’s test in GraphPad Prism 7.0. Transcriptome data together with clinical characteristics were downloaded from TCGA database and processed with R version 4.0.1. Independent t-tests were used to assess the difference of gene expression between tumor tissues and adjacent normal tissues. Kaplan–Meier curve and log-rank test were usually performed to analyze the survival probability between groups based on differences in gene expression. All values were expressed as mean ± SD. *p* values <0.05 indicate statistically significant.

## Results

### The Comparison of Anti-Proliferative Effect Between Paroxetine and Fluoxetine in Multiple Cell Lines

**FIGURE 1:**
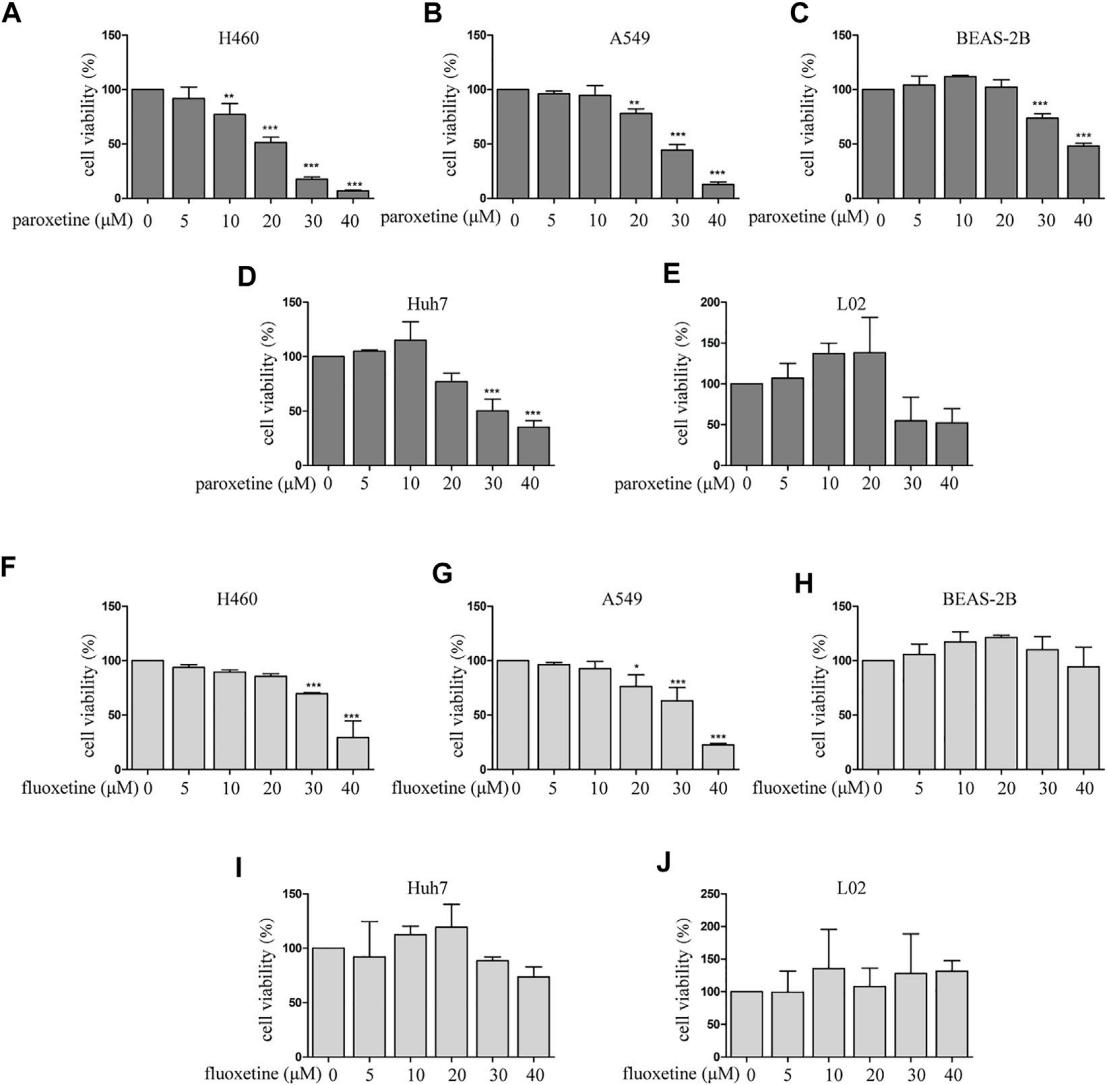
Comparison of paroxetine and fluoxetine for anti-proliferation effect in multiple cell lines. **(A–E)** Lung cancer cells (H460 and A549), normal lung epithelial cells (BEAS-2B), human hepatoma cell line (Huh7), and normal liver cell line (L02) were treated with paroxetine (0, 5, 10, 20, 30, and 40 μM) for 24 h. CCK8 was used to measure cell viability. **(F–J)** H460, A549, BEAS-2B, Huh7, L02 cells were treated with fluoxetine (0, 5, 10, 20, 30, and 40 μM) for 24 h, CCK8 was used to measure cell viability. Data are presented as mean ± SD; *p < 0.05, **p < 0.01, ***p < 0.001.

To explore the beneficial therapeutic strategies for cancer patients with depression, we used antidepressants as a breakthrough to evaluate the therapeutic potential of different drugs. The anti-proliferative activities of paroxetine and fluoxetine in multiple cell types were assessed by CCK8. For NSCLC (non–small cell lung cancer) cell lines (H460 and A549), the results showed that both paroxetine and fluoxetine inhibited the cell viability in a concentration-dependent way (Figures 1A, B, F, G). Then, we compared the cytotoxicity of drugs in the human hepatoma cell line (Huh7). The data showed that paroxetine inhibited the growth of Huh7, while fluoxetine had a slightly inhibitory effect (Figures 1D, I). The results of normal lung epithelial cells (BEAS-2B) and normal liver cell line (L02) indicated that paroxetine had a greater effect on normal cells than fluoxetine (Figures 1C, E, H, J). Based on these data, we thought that the protective effect of fluoxetine on normal cells made it safer in clinical therapies, although the inhibitory effect of fluoxetine on tumor cells was not as significant as that of paroxetine, so we chose fluoxetine for further study.

### Fluoxetine Induced Apoptosis and Arrested Cell Cycle at G0/G1

**FIGURE 2:**
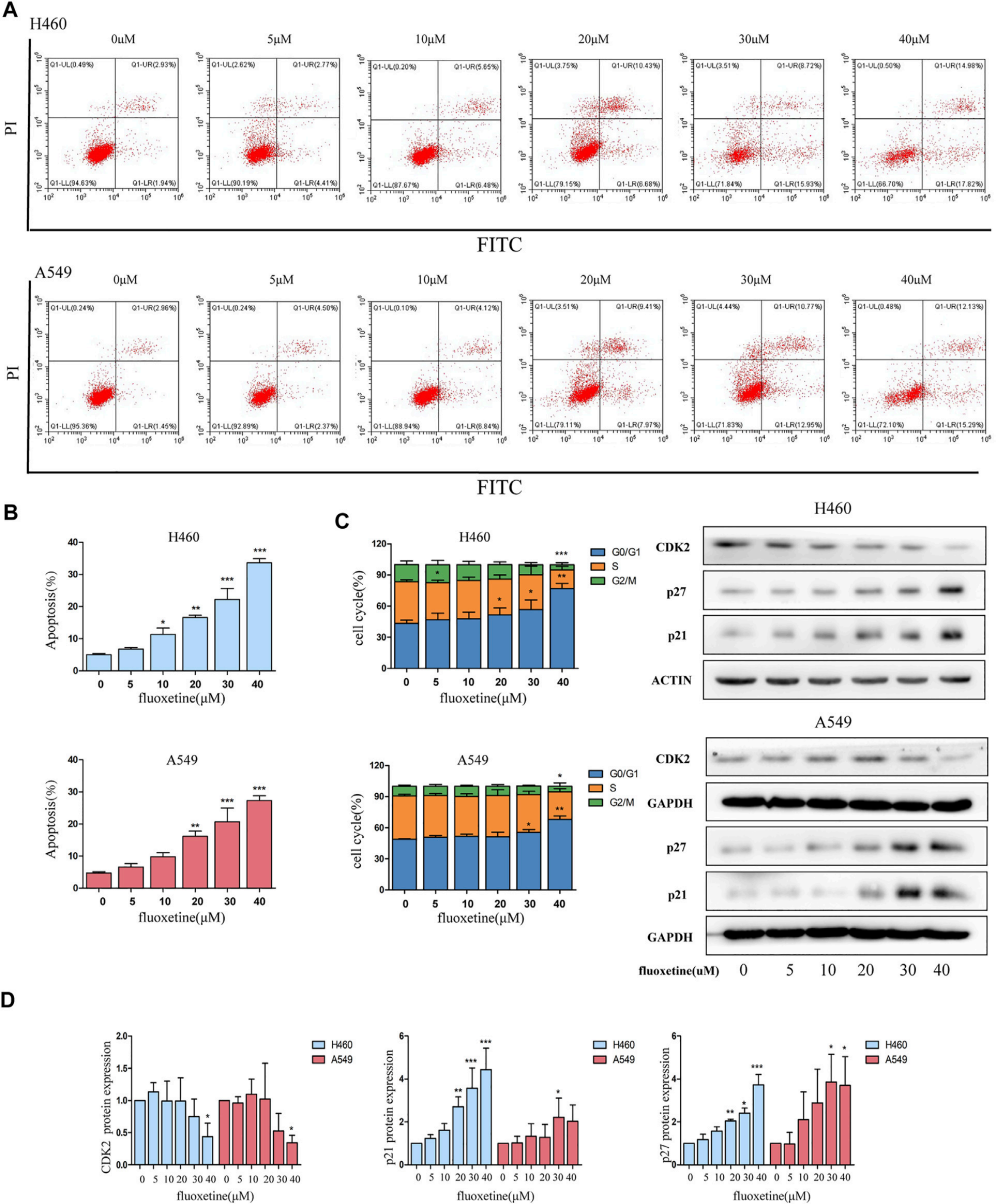
Fluoxetine arrested cell cycle at G0/G1. **(A)** Cells were treated with fluoxetine (0–40 μM) for 24 h, performed PI/FITC staining, and analyzed with flow cytometry. **(B)** Percentage of apoptosis cells was analyzed. **(C)** Distribution of the cell cycle was analyzed by ModFit LT 5.0 software and the result of the Western blot of CDK2, p27, and p21. The images were collected from different parts of the same gel. **(D)** Quantitative analysis of optical band densitometry. Data are presented as mean ± SD; *p < 0.05, **p < 0.01, ***p < 0.001.

To clarify the inhibitory mechanism of fluoxetine, we then focused on the cell apoptosis and cell cycle. Flow cytometry was used to evaluate the percentage of apoptosis and the distribution of the cell cycle. Firstly, we found that fluoxetine could induce apoptosis (Figure 2A). The results of flow cytometry showed that an increase in the percentage of apoptotic cells after fluoxetine treatment (Figure 2B). Then, we observed that the percentage of the G0/G1 phase was increased with the drug concentration, these results indicated that fluoxetine could arrest the cell cycle in G0/G1 phase, and this ability was in a concentration-dependent manner (Figure 2C). Meanwhile, we used western blot to evaluate the expression of cyclin-dependent kinases (CDKs) and p21, p27, the proteins related to the G1 phase. CDKs are the main regulator of the cell cycle, and CDK2 is an essential kinase for the G1/S transition (Du et al., 2016). p21 and p27 are well-known inhibitors of CDK2 (Levkau et al., 1998), which can cause cell cycle arrest at the G0/G1 phase. Our research found that fluoxetine could increase the expression of p21 and p27 and decrease CDK2 in a dose-dependent manner both in the H460 cells and A549 cells (Figure 2D). These results were consistent with the flow cytometry analysis. Therefore, we speculated that fluoxetine might arrest the cell cycle. Altogether, the fluoxetine induced inhibitory influence on cell proliferation was demonstrated from these results.

### Fluoxetine Induced Autophagy

**FIGURE 3:**
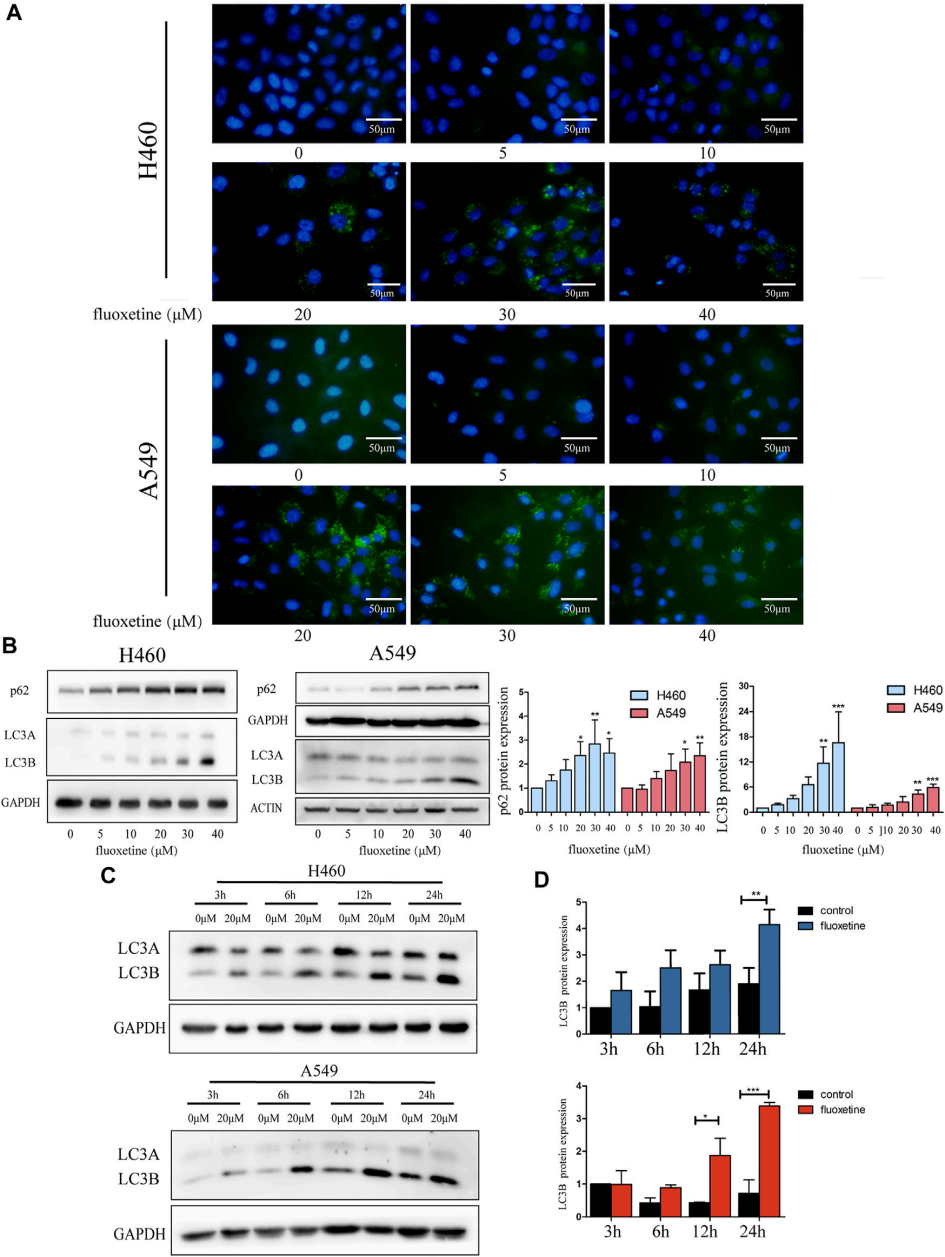
Fluoxetine induced autophagy in a dose and time-dependent way **(A)** Cells were treated with fluoxetine (0–40 μM) for 24 h. Immunofluorescence of LC3 (green) and nuclear (blue) were used to observe autophagy. Scale bars were 50 μm. **(B)** The Western blot result of p62 and LC3. Cells were treated with fluoxetine (0–40 μM) for 24 h. The images were collected from different parts of the same gel. **(C)** Western blot result of LC3 in control and fluoxetine groups. Cells were treated with fluoxetine (20 μM) for 3, 6, 12, and 24 h. The images were collected from different parts of the same gel. **(D)** Quantitative analysis of optical band densitometry. Data are presented as mean ± SD; *p < 0.05, **p < 0.01, ***p < 0.001.

Then, we shone a light on autophagy to discover the underlying mechanism of fluoxetine in regressing cell proliferation. Immunofluorescence was used to observe the autophagosome. We found that the intensity of the fluorescence increased with the drug concentration. The same phenomena could be detected in A549 cells as well (Figure 3A). Then, the western blot was used to measure the expression of autophagy-related proteins. p62 and LC3 are the two markers of autophagy; therefore, these proteins can represent autophagy to some extent. We found that as the drug concentration increased, the expression of p62 and LC3B continued to increase, while the level of LC3A decreased (Figure 3B). Another obvious phenomenon that could be observed was that the induction of autophagy flux gradually became more significant over time. Compared with control group, the level of LC3B changed from 3 h and gradually became obvious, reaching the maximum at 24 h (Figures 3C, D). Therefore, we thought autophagy could be induced by fluoxetine in a dose-dependent and time-dependent fashion.

**FIGURE 4:**
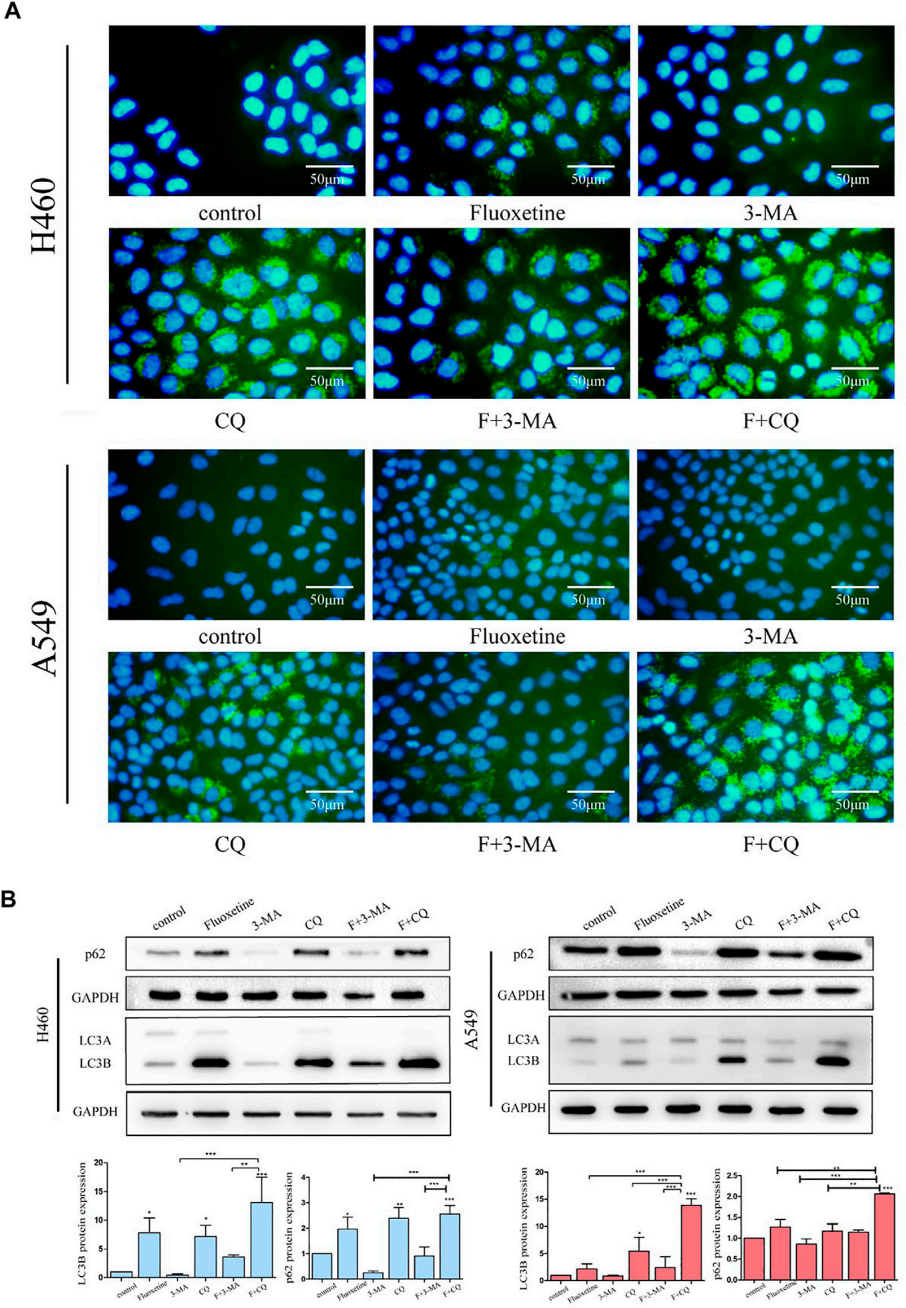
Fluoxetine induced autophagy in a dose and time-dependent way **(A)** immunofluorescence of LC3 (green) and DAPI (blue) was used to observe the autophagy. Cells were treated with 20 μM fluoxetine (F), 1 mM 3-MA, 40 μM CQ, the combination of fluoxetine plus rapamycin, and fluoxetine plus chloroquine for 24 h. Scale bars 50 μm. **(B)** Result of the Western blot of LC3 and p62. The treatment of drugs was used as described previously. Quantitative analysis of optical band densitometry was conducted. Data are presented as mean ± SD; *p < 0.05, **p < 0.01, ***p < 0.001.

3-methyladenine (3-MA) and chloroquine (CQ) are the well-known inhibitors of autophagy, which can prevent the formation of autophagy. Therefore, we used 3-MA and CQ to further investigate the function of fluoxetine to autophagy. The image of immunofluorescence showed that the treatment of CQ did produce the autophagic flux, and when combined with fluoxetine, the phenomenon was more obvious. In contrast, using 3-MA for 24 h led to decreased fluoxetine-induced LC3B formation (Figure 4A). Then, we applied western blot to further confirm this result. The data demonstrated that fluoxetine or CQ alone increased the expression of LC3B and p62, and the co-treatment of fluoxetine and CQ could produce higher expression. When using 3-MA, the expression of LC3B and p62 were low. Both effects occurred on H460 and A549 cells (Figure 4B). These results confirmed that fluoxetine had a role in the induction of autophagy.

### Fluoxetine had a Connection With ER Stress and the mTOR Signaling Pathway

**FIGURE 5:**
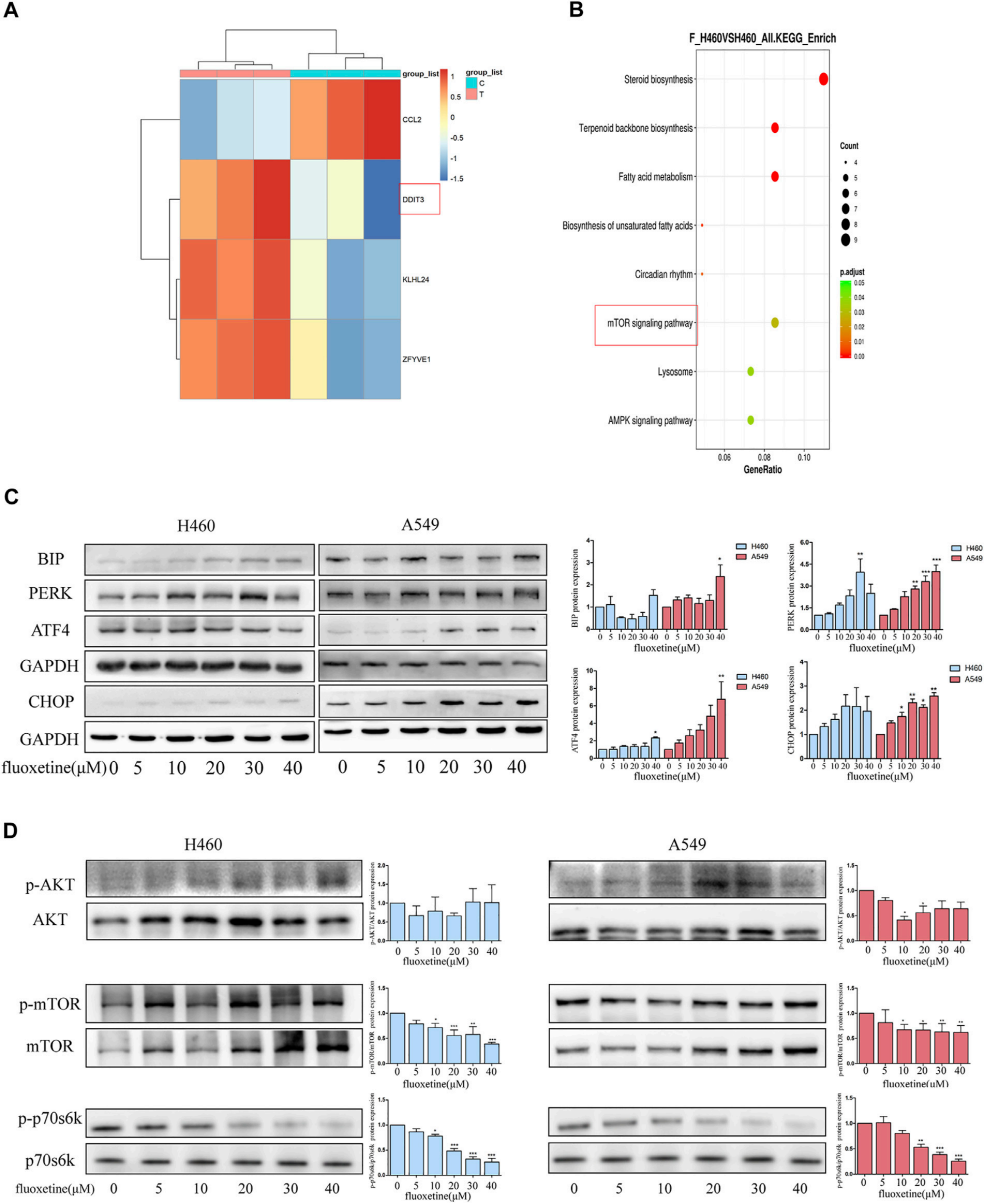
Fluoxetine had a connection with ER stress and mTOR signaling pathway. **(A)** Autophagy-related genes were analyzed in a gene set. **(B)** Top 8 enriched KEGG pathways. **(C)**Western blot was used to analyze the expression ER stress related proteins, including BIP, PERK, ATF4, and CHOP. The images were collected from different parts of the same gel. **(D)** Western blot was used to analyze the expression of p-AKT, AKT, p-mTOR, mTOR, p-p70s6k, and p70s6k. The images were collected from different parts of the same gel. Data are presented as mean ± SD; *p < 0.05, **p < 0.01, ***p < 0.001.

In order to investigate the specific mechanism of fluoxetine, we chose H460 cells that were more sensitive to the drug. RNA sequencing (RNA-seq) was used to study the transcriptome of cells treated with fluoxetine and analyze the difference with control treatment. In this result, 166 differentially expressed genes (*p* < 0.05, fold change ≥2) were identified, of which 12 genes were downregulated, and 154 genes were upregulated (Supplementary Figure S1). We further analyzed the autophagy-related genes in the gene pool, and four genes proved to be significantly different. Interestingly, we found DDIT3, also known as C/EBP homologous protein (CHOP), was upregulated after fluoxetine treatment (Figure 5A). CHOP is a characteristic biomarker of ER stress (Senft and Ronai, 2015). A plethora of studies has been conducted to investigate the relationship between ER stress and autophagy (Song et al., 2018). Taking these into consideration, we wanted to explore the connection between autophagy and ER stress after fluoxetine treatment. Western blot was used to detect the ER stress-related markers. BIP expression was detected firstly, and it was found that fluoxetine induced an up-regulation of BIP levels. The activation of BIP can trigger the downstream sensors and the PERK pathway involved in autophagy is one of these sensors (Kouroku et al., 2007). The result of western blot showed that fluoxetine increased the content of PERK, activating transcription factor 4 (ATF4), and CHOP in a dose-dependent way (Figure 5C). All these results indicate that fluoxetine had a close relationship with ER stress.

The analysis of the KEGG showed that the genes were enriched in 8 pathways which included biosynthesis, metabolism, mechanistic target of rapamycin kinase (mTOR) signaling pathway, lysosome, and AMPK signaling pathway (Figure 5B). AKT serine/threonine kinase (AKT)/mTOR signaling pathway has been proved to play a critical role in regulating the process of autophagy (Heras-Sandoval et al., 2014). Hence, we detected the expression of AKT and mTOR. In our results, the fluoxetine could down-regulate the p-AKT/AKT, p-mTOR/mTOR, and p-p70s6k/p70s6k in a dose-dependent way (Figure 5D). Based on these results, we thought that the ER stress and AKT/mTOR signaling pathway might play a significant role in fluoxetine-treated cells.

### AKT/mTOR Signaling Pathway was Regulated Through ATF4 in Fluoxetine Treatment

**FIGURE 6:**
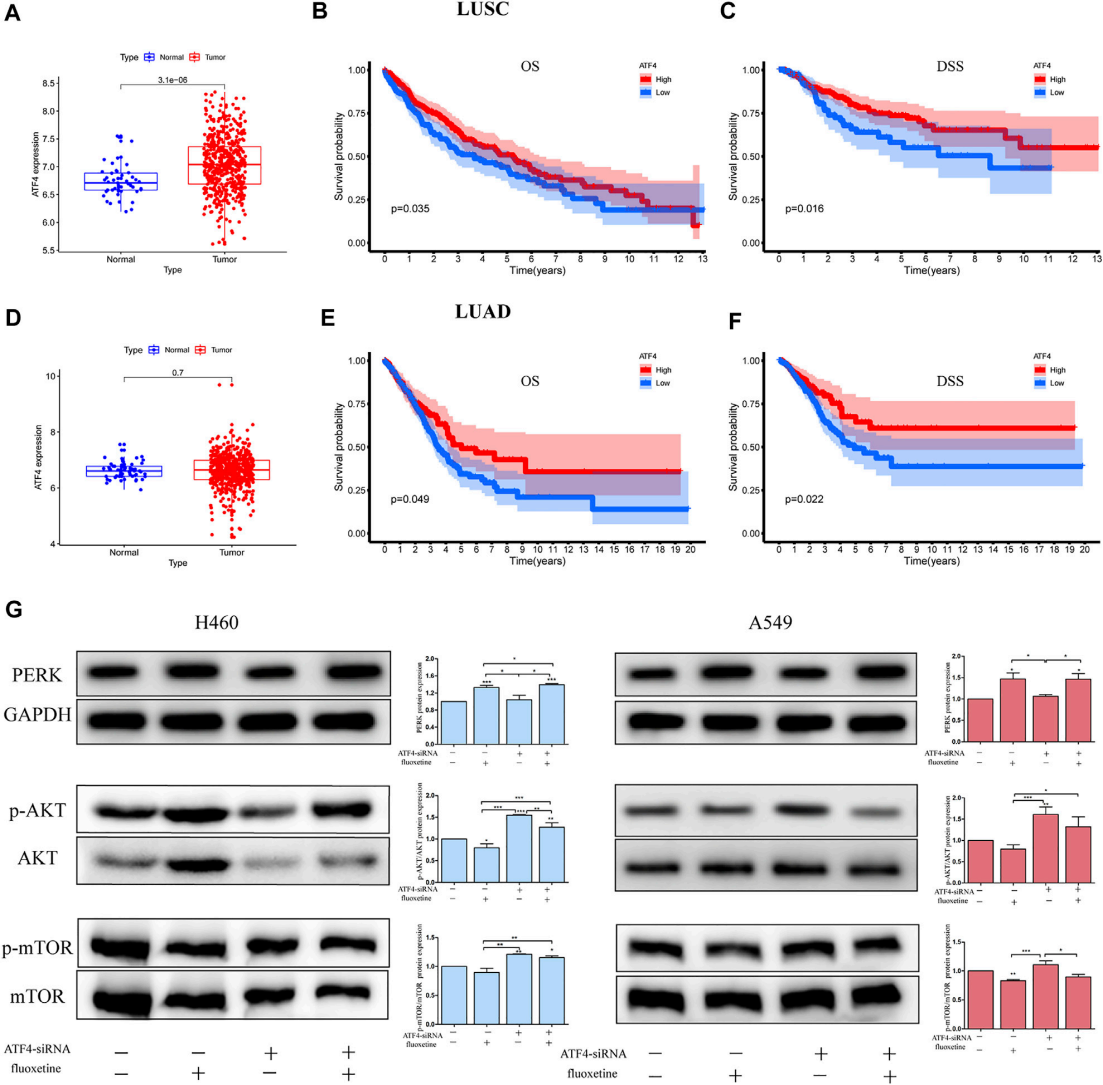
AKT/mTOR signaling pathway was regulated through ATF4 in fluoxetine treatment. **(A,D)** Expression of ATF4 in normal and tumor tissues was detected in the TCGA database. The blue spots on the left were the normal group, and the red spots on the right were the tumor group. [**(B,C)**, **(E,F)**] Survival analysis comparing high to low expression of ATF4 in lung cancer. **(G)** Expression of PERK, p-AKT/AKT and p-mTOR/mTOR in lung cancer cells transfected with ATF4 siRNA for 48 h followed by exposure to fluoxetine (20 μM) for another 24 h was tested by Western blot. The images were collected from different parts of the same gel. Data are presented as mean ± SD; *p < 0.05, **p < 0.01, ***p < 0.001.

A recent study has demonstrated that ER stress can regulate the mTOR signaling pathway to exert anti-cancer effect (Yao et al., 2020). Thus, we further investigated the regulation of ER stress and AKT/mTOR signaling pathway induced by fluoxetine. The role of CHOP and ATF4 were identified first. The bioinformatic analysis showed that ATF4 had a higher expression in the tissues of patients with lung squamous cell carcinoma (LUSC) and lung adenocarcinoma (LUAD) (Figures 6A, D), and the higher expression of ATF4 was significantly related to longer overall survival (OS) and disease-specific survival (DSS) of NSCLC patients (Figures 6B, C, E, F). The role of CHOP in expression and prognosis is not obvious (data not shown). Therefore, we chose ATF4 for further experimental validation. To clarify whether the downregulation of AKT/mTOR signaling pathway was induced by ATF4, the cells were transfected with ATF4 siRNA and the western blot was used to examine the efficiency of siRNA (Supplementary Figure S2). We then investigated whether fluoxetine-mediated inhibition of AKT/mTOR occurred through increased ATF4. The cells were transfected with ATF4 siRNA, and levels of PERK and p-AKT/AKT and p-mTOR/mTOR were assessed after fluoxetine treatment. Compared with NC-siRNA, the expression of p-AKT/AKT and p-mTOR/mTOR recovered in ATF4-knockdown cells, and the expression was reduced after fluoxetine treatment. The expression of PERK was unchanged with transfected with ATF4 siRNA. (Figure 6G).

### The Fluoxetine-Induced Anticancer Effect Through the ATF4-AKT-mTOR Signaling Pathway

**FIGURE 7:**
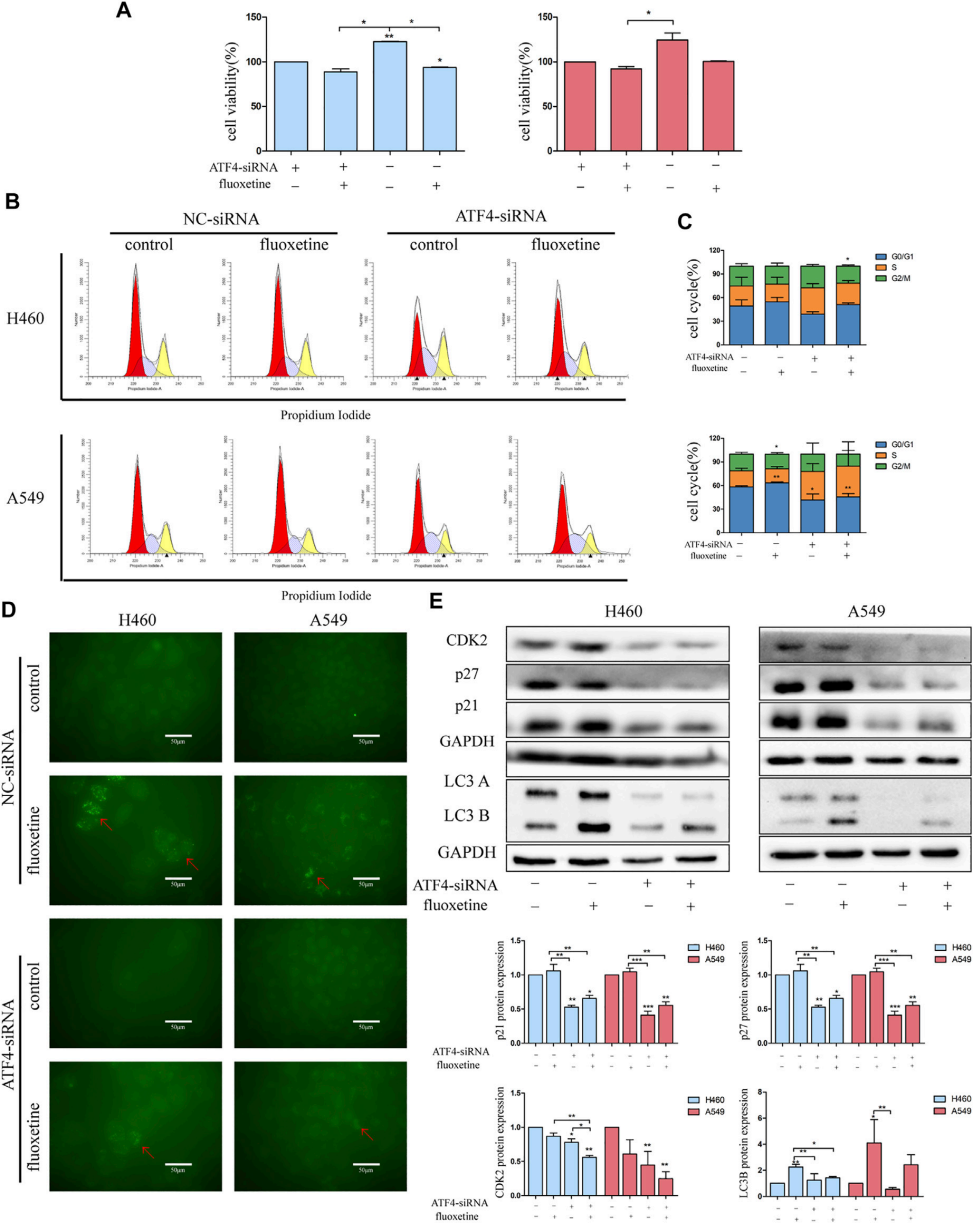
Fluoxetine-induced anticancer effect through the ATF4-AKT-mTOR signaling pathway **(A)** the cell viability in lung cancer cells transfected with ATF4 siRNA for 48 h followed by exposure to fluoxetine (20 μM) for another 24 h was assessed by CCK8. Left and right indicate H460 cells and A549 cells, respectively. **(B,C)** The distribution of the cell cycle in lung cancer cells transfected with ATF4 siRNA for 48 h followed by exposure to fluoxetine (20 μM) for another 24 h was analyzed by flow cytometry. **(D)** The autophagy in lung cancer cells transfected with ATF4 siRNA for 48 h followed by exposure to fluoxetine (20 μM) for another 24 h was observed by immunofluorescence of LC3 (green). Scale bars were 50 μm. **(E)** The expression of CDK2, p27, p21, and LC3 in lung cancer cells transfected with ATF4 siRNA for 48 h followed by exposure to fluoxetine (20 μM) for another 24 h was tested by Western blot. Left and right indicate H460 cells and A549 cells, respectively. The images were collected from different parts of the same gel. Data are presented as mean ± SD; *p < 0.05, **p < 0.01, ***p < 0.001.

To analyze whether the fluoxetine induced anticancer effect through the ATF4-AKT-mTOR signaling pathway, cell proliferation, apoptosis, cell cycle, and autophagy were measured after ATF4 siRNA treatment. The CCK8 assay showed that the cell viability recovered after ATF4 knockdown (Figure 7A). The analysis of cell cycle results found that the proportion of G0/G1 phase and the expression of G0/G1 related proteins were reduced after ATF4 knockdown (Figures 7B, C, E). Moreover, the results of immunofluorescence and protein level of LC3B further confirmed that the treatment with ATF4 siRNA decreased the induction of autophagy after fluoxetine treatment (Figures 7D, E). All these data indicated that the ATF4-AKT-mTOR signaling pathway exerted a significant role in the fluoxetine-induced anticancer effect. Surprisingly, the results of apoptosis showed that the percentage of apoptosis after ATF4 knockdown were not statistically significant. This phenomenon means that fluoxetine induced apoptosis might not be through ATF4-AKT-mTOR signaling pathway (Supplementary Figure S3).

### Fluoxetine Exerted Anti-Tumor Effects While Not Damaging Normal Cells

**FIGURE 8:**
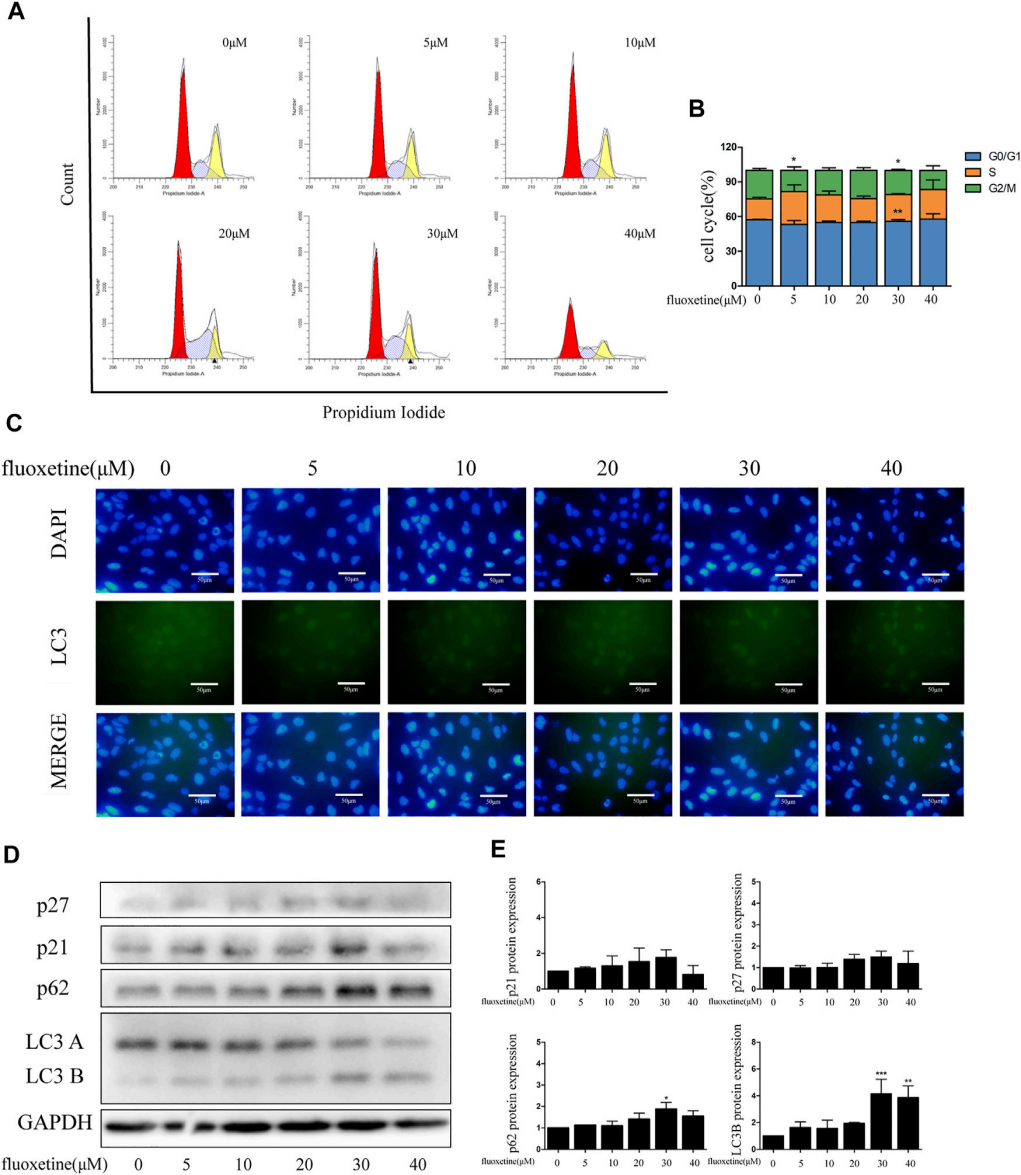
Fluoxetine exerted anti-tumor effects while not damaging normal cells. **(A–B)** Distribution of the cell cycle in normal lung epithelial cells treated with fluoxetine (0–40 μM) was analyzed by flow cytometry. **(C)** Immunofluorescence of LC3 (green) and DAPI (blue) was used to observe the autophagy. Scale bars were 50 μm. **(D–E)** Expression of p27, p21, p62, and LC3 was tested by Western blot. The images were collected from different parts of the same gel. Data are presented as mean ± SD; *p < 0.05, **p < 0.01, ***p < 0.001.

The previous result showed that fluoxetine had no inhibitory effect on normal lung epithelial cells. To confirm this result further, we used flow cytometry and immunofluorescence to analyze. The results demonstrated that fluoxetine was unacted on the cell cycle and autophagy (Figures 8A–C). Western blot also revealed that the protein levels of cell cycle and autophagy were slightly changed after fluoxetine treatment (Figures 8D, E).

## Discussion

Depression is frequently found in the cancer patients, with higher prevalence than general population (Bortolato et al., 2017). Antidepressants are the common drugs to cure depression and also have the anticancer effect (Hsu et al., 2020). However, different antidepressants may have different impact on tumors. In our research, we discovered that fluoxetine inhibited the cell proliferation and induced autophagy of lung cancer cells H460 and A549 cells. Compared with paroxetine, we found that fluoxetine specifically inhibited the proliferation of H460 and A549 cells without influencing liver cancer cell Huh7. Then, we found that fluoxetine upregulated the proportion of G0/G1 phase and affected cell cycle-related proteins, which were consistent with the previous findings (Chen et al., 2019; Marcinkute et al., 2019).

In general, the existing anticancer drugs not only target at cancer cells but also normal cells, which will cause serious side effects, including hair loss, neurotoxicity, and cardiotoxicity (Singh et al., 2016). Therefore, developing ideal anticancer drugs that specifically target cancer cells is of great significance. More interestingly and importantly, we first found that fluoxetine hardly affected BEAS-2B. In our results, fluoxetine has less toxicity in normal lung epithelial cell.

Autophagy is a conserved catabolic process that is induced by stresses and cellular signals. The process of autophagy contains the production of double-membraned vesicles, which engulf the cellular components, forming the complex called autophagosomes (Lebovitz et al., 2015). To get further insights into the mechanisms and targets of the fluoxetine anti-tumor activity in lung cancer cells, we found that fluoxetine induced autophagy. For the first time paying attention to the autophagy induced by fluoxetine in lung cancer cells, we further detect the expression of autophagy-related proteins. P62, also called sequestosome-1 (SQSTM1), is a crucial regulator in autophagy (Liu et al., 2016). p62 interacts with LC3 to form autophagosome, ultimately fusing with lysosome and degradation (Pankiv et al., 2007). Therefore, the level of p62 can reflect the activity of lysosomes. In our study, both p62 and LC3B increased with fluoxetine concentrations. We further explored the effect of fluoxetine on autophagy. The results of Immunofluorescence and western blot showed that CQ increased the level of LC3B and p62 after combined with fluoxetine, and 3-MA decreased the expression. Autophagy can be regulated by affecting the formation of autophagosome and subsequent degradation. Therefore, the accumulation of autophagy may represent the induction of autophagy or inhibition of degradation. LC3 turn over assay demonstrated that the change in the amount of LC3 between the absence and presence of lysosome inhibitor indicates the amount of LC3 degradation in the lysosome (Mizushima et al., 2010). In the present study, the significant accumulation of LC3B in the CQ and fluoxetine group meant that fluoxetine had a role in the induction of autophagy. The increased expression of p62 in the CQ combined with fluoxetine group reflected that the presence of autophagy-dependent degradation of p62 in the fluoxetine treatment. Thus, in our study, we speculated that fluoxetine could induce autophagy, but it also destroyed the function of lysosomes to a certain extent, which led to an abnormal increase of p62.

The result of RNA-seq hinted that the CHOP had a connection with autophagy. CHOP is a marker of UPR, and UPR is an adaptive response to ER stress. Previous studies showed that some drugs can continuously induce ER stress through the UPR pathway to exert anticancer effects, which makes UPR a potential target for anti-cancer therapy (Lin et al., 2019). There are points of similarity between UPR and autophagy in cancer treatment, with low levels of activation being protective and high levels of inducing cell death. A link between UPR and autophagy has been clarified. Under ER stress, the induction of autophagy relied on the PERK–eIF2α pathway (B’Chir et al., 2013). PERK is an essential sensor for translation regulation, which increases the expression of ATF4 and CHOP during the URP (Harding et al., 2000). Moreover, the PERK-ATF4 signaling pathway was found to be activated in some cancers and the PERK-induced autophagy decreased the damage of ROS accumulation (Atkins et al., 2013). The activation of ATF4 and CHOP further induces the autophagy-related protein such as Atg5, Atg12, and Atg16L. The formation of the Atg5 complex participates in the elongation process of autophagy (B’Chir et al., 2013). We analyzed the ER stress in fluoxetine-treated cells. The UPR-related proteins such as BIP, PERK, ATF4, and CHOP were detected, the levels of these proteins all increased with the increasing concentration, indicting the activation of PERK pathway. Coupled with the result of inhibitors on autophagy-related proteins, we speculate that fluoxetine affects the expansion of autophagy by activating ATF4-CHOP pathway, and the higher expression of LC3B with CQ treatment further confirm this hypothesis. Previous research found that fluoxetine reversed depressive-like behavior in mice through PI3K/Akt/mTOR/p-ERK1/2 signaling pathways (Amin et al., 2020). Coupled with the result of KEGG, we wonder whether AKT/mTOR signaling pathway participated in the fluoxetine treatment. The activation of signaling pathway is a cascade reaction. PI3K phosphorylates AKT, and the activated AKT further phosphorylates mTOR. The results of western blot showed that the levels of p-AKT/AKT, p-mTOR/mTOR were significantly decreased, indicating the involvement of the AKT/mTOR pathway. Remarkably, the AKT/mTOR pathway was also related to the activation of ER stress. We now have confirmed that fluoxetine induced ER stress which increased ATF4, this upregulation further inhibited AKT/mTOR signaling pathway.

**FIGURE 9:**
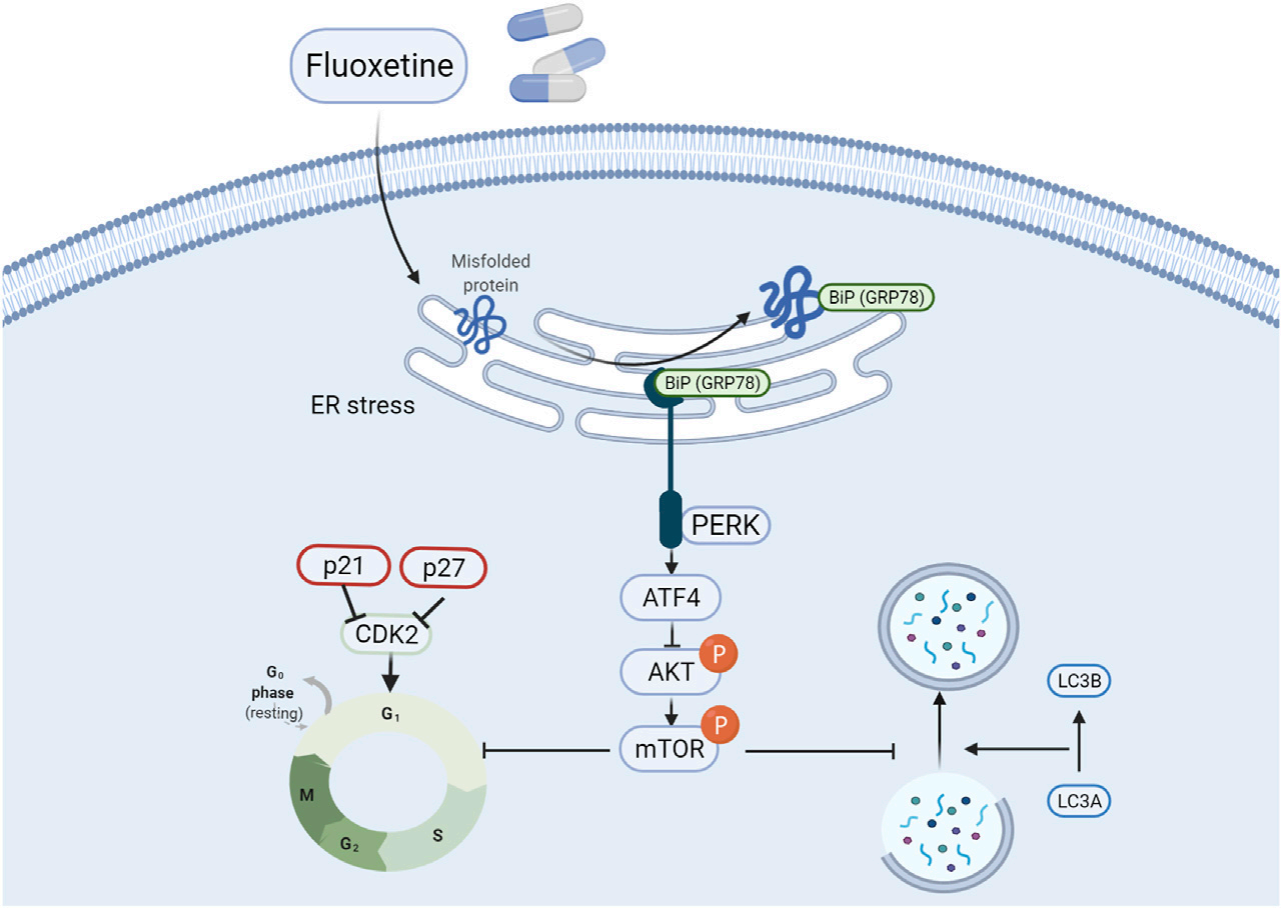
Fluoxetine induced cell cycle arrest and autophagy by triggering ATF4-AKT-mTOR signaling pathway in lung cancer cells. Briefly, fluoxetine activated the ER stress related proteins and the increased ATF4 inactivated the AKT/mTOR signaling pathway, thus leading to the cell cycle arrest at G0/G1 phase and autophagy.

To our knowledge, there is no report on the study of the relationship between fluoxetine with ER stress and anticancer effect in lung cancer cells. So, it is of great importance to discover the involvement of the ATF4-AKT-mTOR signaling pathway in the anti-tumor activity of fluoxetine in lung cancer cells. By regulating p21 and p27, the AKT/mTOR influenced the cell cycle to change cell proliferation. In addition, mTOR has been recognized as a critical regulator of autophagy (Shi et al., 2019), and the AKT/mTOR pathway has also been widely reported to be related to autophagy in neurodegeneration (Heras-Sandoval et al., 2014) and cancer treatment (Xu et al., 2020). In line with these results, we further confirmed that the fluoxetine could activate the ATF4-AKT-mTOR pathway to induce cell cycle arrest and autophagy to restraint cancer cells’ growth without affecting normal cells (Figure 9).

Although it has been confirmed that fluoxetine has a potential anticancer effect, its molecular targets in certain cancers are quite unclear. Compared with previous reports, our results elaborate the molecular mechanism of fluoxetine in lung cancer. However, this study only proved the limited phenotypes induced by the ATF4-AKT-mTOR pathway after fluoxetine treatment, and further researches need to explore the broader functions of fluoxetine and investigate the influence of fluoxetine on multiple cell types, such as immunocytes. And based on the different effect of fluoxetine between normal cells and cancer cells, our future work will focus on the early tumor prevention with the use of fluoxetine and explore whether timely use of fluoxetine can alleviate the development of tumors in patients with depression.

## Conclusion

In conclusion, we discovered that fluoxetine exerted an anti-proliferation role in non–small lung cancer cells and induced cell cycle arrest, ER stress and autophagy by triggering ATF4-AKT-mTOR signaling pathway. More importantly, the cell-killing effect of fluoxetine had no influence on normal cells, which made it safer in the clinical therapy of lung cancer patients with depression.

## Data Availability

The datasets presented in this study can be found in online repositories. The names of the repository/repositories and accession number(s) can be found below: https://www.ncbi.nlm.nih.gov/geo/; GSE200209.

## References

[B1] Akechi T. Okuyama T. Onishi J. Morita T. Furukawa T. A. (2008). Psychotherapy for Depression Among Incurable Cancer Patients. Cochrane Database Syst. Rev. 2008 (2), CD005537. 10.1002/14651858.CD005537.pub2 PMC6464138 18425922

[B2] Amin N. Xie S. Tan X. Chen Y. Ren Q. Botchway B. O. A. (2020). Optimized Integration of Fluoxetine and 7, 8-dihydroxyflavone as an Efficient Therapy for Reversing Depressive-like Behavior in Mice during the Perimenopausal Period. Prog. Neuropsychopharmacol. Biol. Psychiatry 101, 109939. 10.1016/j.pnpbp.2020.109939 32243998

[B3] Atkins C. Liu Q. Minthorn E. Zhang S. Y. Figueroa D. J. Moss K. (2013). Characterization of a Novel PERK Kinase Inhibitor with Antitumor and Antiangiogenic Activity. Cancer Res. 73, 1993–2002. 10.1158/0008-5472.CAN-12-3109 23333938

[B4] B'Chir W. Maurin A. C. Carraro V. Averous J. Jousse C. Muranishi Y. (2013). The eIF2α/ATF4 Pathway Is Essential for Stress-Induced Autophagy Gene Expression. Nucleic Acids Res. 41, 7683–7699. 10.1093/nar/gkt563 23804767 PMC3763548

[B5] Bertolotti A. Zhang Y. Hendershot L. M. Harding H. P. Ron D. (2000). Dynamic Interaction of BiP and ER Stress Transducers in the Unfolded-Protein Response. Nat. Cel Biol. 2, 326–332. 10.1038/35014014 10854322

[B6] Bettigole S. E. Glimcher L. H. (2015). Endoplasmic Reticulum Stress in Immunity. Annu. Rev. Immunol. 33, 107–138. 10.1146/annurev-immunol-032414-112116 25493331

[B7] Bortolato B. Hyphantis T. N. Valpione S. Perini G. Maes M. Morris G. (2017). Depression in Cancer: The many Biobehavioral Pathways Driving Tumor Progression. Cancer Treat. Rev. 52, 58–70. 10.1016/j.ctrv.2016.11.004 27894012

[B8] Chen W. Zheng R. Baade P. D. Zhang S. Zeng H. Bray F. (2016). Cancer Statistics in China, 2015. CA Cancer J. Clin. 66, 115–132. 10.3322/caac.21338 26808342

[B9] Chen W. T. Hsu F. T. Liu Y. C. Chen C. H. Hsu L. C. Lin S. S. (2019). Fluoxetine Induces Apoptosis through Extrinsic/Intrinsic Pathways and Inhibits ERK/NF-κB-Modulated Anti-apoptotic and Invasive Potential in Hepatocellular Carcinoma Cells *In Vitro* . Int. J. Mol. Sci. 20 (3), 757. 10.3390/ijms20030757 PMC6386946 30754643

[B10] Dey S. Sayers C. M. Verginadis I. I. Lehman S. L. Cheng Y. Cerniglia G. J. (2015). ATF4-dependent Induction of Heme Oxygenase 1 Prevents Anoikis and Promotes Metastasis. J. Clin. Invest. 125, 2592–2608. 10.1172/JCI78031 26011642 PMC4563676

[B11] Du W. W. Yang W. Liu E. Yang Z. Dhaliwal P. Yang B. B. (2016). Foxo3 Circular RNA Retards Cell Cycle Progression via Forming Ternary Complexes with P21 and CDK2. Nucleic Acids Res. 44, 2846–2858. 10.1093/nar/gkw027 26861625 PMC4824104

[B12] Ginsburg A. (2008). Cancer-related Depression and Potential Pharmacologic Therapies. Proc (Bayl Univ Med Cent) 21, 439–441. 10.1080/08998280.2008.11928449 18982092 PMC2566922

[B13] Harding H. P. Zhang Y. Bertolotti A. Zeng H. Ron D. (2000). Perk Is Essential for Translational Regulation and Cell Survival during the Unfolded Protein Response. Mol. Cel. 5, 897–904. 10.1016/s1097-2765(00)80330-5 10882126

[B14] Heras-Sandoval D. Pérez-Rojas J. M. Hernández-Damián J. Pedraza-Chaverri J. (2014). The Role of PI3K/AKT/mTOR Pathway in the Modulation of Autophagy and the Clearance of Protein Aggregates in Neurodegeneration. Cell. Signal. 26, 2694–2701. 10.1016/j.cellsig.2014.08.019 25173700

[B15] Hopwood P. Stephens R. J. (2000). Depression in Patients with Lung Cancer: Prevalence and Risk Factors Derived from Quality-Of-Life Data. J. Clin. Oncol. 18, 893–903. 10.1200/JCO.2000.18.4.893 10673533

[B16] Hsu L. C. Tu H. F. Hsu F. T. Yueh P. F. Chiang I. T. (2020). Beneficial Effect of Fluoxetine on Anti-tumor Progression on Hepatocellular Carcinoma and Non-small Cell Lung Cancer Bearing Animal Model. Biomed. Pharmacother. 126, 110054. 10.1016/j.biopha.2020.110054 32145588

[B17] Jang J. H. Kim Y. J. Kim H. Kim S. C. Cho J. H. (2015). Buforin IIb Induces Endoplasmic Reticulum Stress-Mediated Apoptosis in HeLa Cells. Peptides 69, 144–149. 10.1016/j.peptides.2015.04.024 25958204

[B18] Janssens S. Pulendran B. Lambrecht B. N. (2014). Emerging Functions of the Unfolded Protein Response in Immunity. Nat. Immunol. 15, 910–919. 10.1038/ni.2991 25232821 PMC4388558

[B19] Kouroku Y. Fujita E. Tanida I. Ueno T. Isoai A. Kumagai H. (2007). ER Stress (PERK/eIF2alpha Phosphorylation) Mediates the Polyglutamine-Induced LC3 Conversion, an Essential Step for Autophagy Formation. Cell Death Differ 14, 230–239. 10.1038/sj.cdd.4401984 16794605

[B20] Lebovitz C. B. Devorkin L. Bosc D. Rothe K. Singh J. Bally M. (2015). Precision Autophagy: Will the Next Wave of Selective Autophagy Markers and Specific Autophagy Inhibitors Feed Clinical Pipelines? Autophagy 11, 1949–1952. 10.1080/15548627.2015.1078962 26506897 PMC4824567

[B21] Lee C. S. Kim Y. J. Jang E. R. Kim W. Myung S. C. (2010). Fluoxetine Induces Apoptosis in Ovarian Carcinoma Cell Line OVCAR-3 through Reactive Oxygen Species-dependent Activation of Nuclear Factor-kappaB. Basic Clin. Pharmacol. Toxicol. 106, 446–453. 10.1111/j.1742-7843.2009.00509.x 20050848

[B22] Levkau B. Koyama H. Raines E. W. Clurman B. E. Herren B. Orth K. (1998). Cleavage of p21Cip1/Waf1 and p27Kip1 Mediates Apoptosis in Endothelial Cells through Activation of Cdk2: Role of a Caspase cascade. Mol. Cel. 1, 553–563. 10.1016/s1097-2765(00)80055-6 9660939

[B23] Lin Y. Jiang M. Chen W. Zhao T. Wei Y. (2019). Cancer and ER Stress: Mutual Crosstalk between Autophagy, Oxidative Stress and Inflammatory Response. Biomed. Pharmacother. 118, 109249. 10.1016/j.biopha.2019.109249 31351428

[B24] Liu W. J. Ye L. Huang W. F. Guo L. J. Xu Z. G. Wu H. L. (2016). p62 Links the Autophagy Pathway and the Ubiqutin-Proteasome System upon Ubiquitinated Protein Degradation. Cell. Mol. Biol. Lett. 21, 29. 10.1186/s11658-016-0031-z 28536631 PMC5415757

[B25] Marcinkute M. Afshinjavid S. Fatokun A. A. Javid F. A. (2019). Fluoxetine Selectively Induces P53-independent Apoptosis in Human Colorectal Cancer Cells. Eur. J. Pharmacol. 857, 172441. 10.1016/j.ejphar.2019.172441 31181210

[B26] Mitchell A. J. Chan M. Bhatti H. Halton M. Grassi L. Johansen C. (2011). Prevalence of Depression, Anxiety, and Adjustment Disorder in Oncological, Haematological, and Palliative-Care Settings: A Meta-Analysis of 94 Interview-Based Studies. Lancet Oncol. 12, 160–174. 10.1016/S1470-2045(11)70002-X 21251875

[B27] Mizushima N. Yoshimori T. Levine B. (2010). Methods in Mammalian Autophagy Research. Cell 140, 313–326. 10.1016/j.cell.2010.01.028 20144757 PMC2852113

[B28] Pankiv S. Clausen T. H. Lamark T. Brech A. Bruun J. A. Outzen H. (2007). p62/SQSTM1 Binds Directly to Atg8/LC3 to Facilitate Degradation of Ubiquitinated Protein Aggregates by Autophagy. J. Biol. Chem. 282, 24131–24145. 10.1074/jbc.M702824200 17580304

[B29] Senft D. Ronai Z. A. (2015). UPR, Autophagy, and Mitochondria Crosstalk Underlies the ER Stress Response. Trends Biochem. Sci. 40, 141–148. 10.1016/j.tibs.2015.01.002 25656104 PMC4340752

[B30] Shen Y. Yang J. Zhao J. Xiao C. Xu C. Xiang Y. (2015). The Switch from ER Stress-Induced Apoptosis to Autophagy via ROS-Mediated JNK/p62 Signals: A Survival Mechanism in Methotrexate-Resistant Choriocarcinoma Cells. Exp. Cel Res. 334, 207–218. 10.1016/j.yexcr.2015.04.010 25912909

[B31] Shi B. Ma M. Zheng Y. Pan Y. Lin X. (2019). mTOR and Beclin1: Two Key Autophagy-Related Molecules and Their Roles in Myocardial Ischemia/reperfusion Injury. J. Cel. Physiol. 234, 12562–12568. 10.1002/jcp.28125 PMC13484135 30618070

[B32] Singh S. Sharma B. Kanwar S. S. Kumar A. (2016). Lead Phytochemicals for Anticancer Drug Development. Front. Plant Sci. 7, 1667. 10.3389/fpls.2016.01667 27877185 PMC5099879

[B33] Song S. Tan J. Miao Y. Zhang Q. (2018). Crosstalk of ER Stress-Mediated Autophagy and ER-Phagy: Involvement of UPR and the Core Autophagy Machinery. J. Cel. Physiol. 233, 3867–3874. 10.1002/jcp.26137 28777470

[B34] Stepulak A. Rzeski W. Sifringer M. Brocke K. Gratopp A. Kupisz K. (2008). Fluoxetine Inhibits the Extracellular Signal Regulated Kinase Pathway and Suppresses Growth of Cancer Cells. Cancer Biol. Ther. 7, 1685–1693. 10.4161/cbt.7.10.6664 18836303

[B35] Walker M. S. Zona D. M. Fisher E. B. (2006). Depressive Symptoms after Lung Cancer Surgery: Their Relation to Coping Style and Social Support. Psychooncology 15 (8), 684–693. 10.1002/pon.997 16302291

[B36] Xu Z. Han X. Ou D. Liu T. Li Z. Jiang G. (2020). Targeting PI3K/AKT/mTOR-mediated Autophagy for Tumor Therapy. Appl. Microbiol. Biotechnol. 104, 575–587. 10.1007/s00253-019-10257-8 31832711

[B37] Yao W. Lin Z. Shi P. Chen B. Wang G. Huang J. (2020). Delicaflavone Induces ROS-Mediated Apoptosis and Inhibits PI3K/AKT/mTOR and Ras/MEK/Erk Signaling Pathways in Colorectal Cancer Cells. Biochem. Pharmacol. 171, 113680. 10.1016/j.bcp.2019.113680 31669234

