# Supplementary figures and images for: Antidepressants Fluoxetine Mediates Endoplasmic Reticulum Stress and Autophagy of Non–Small Cell Lung Cancer Cells Through the ATF4-AKT-mTOR Signaling Pathway
Source: Front Pharmacol. 2022 May 10;13:904701. doi: 10.3389/fphar.2022.904701 (PMC9127500; doi:10.3389/fphar.2022.904701)

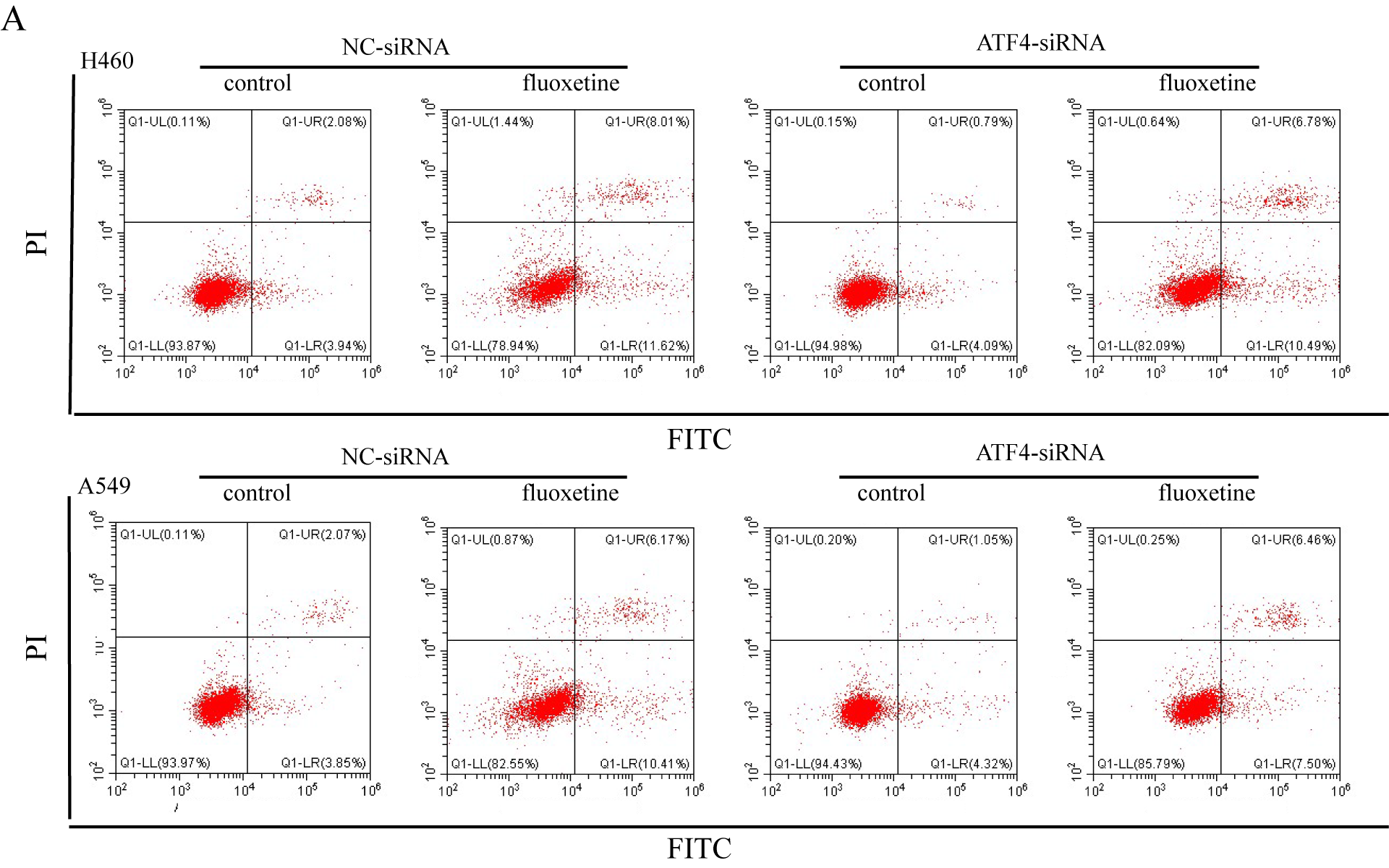

Supplement: Supplementary file 1 [file Image3.TIF]

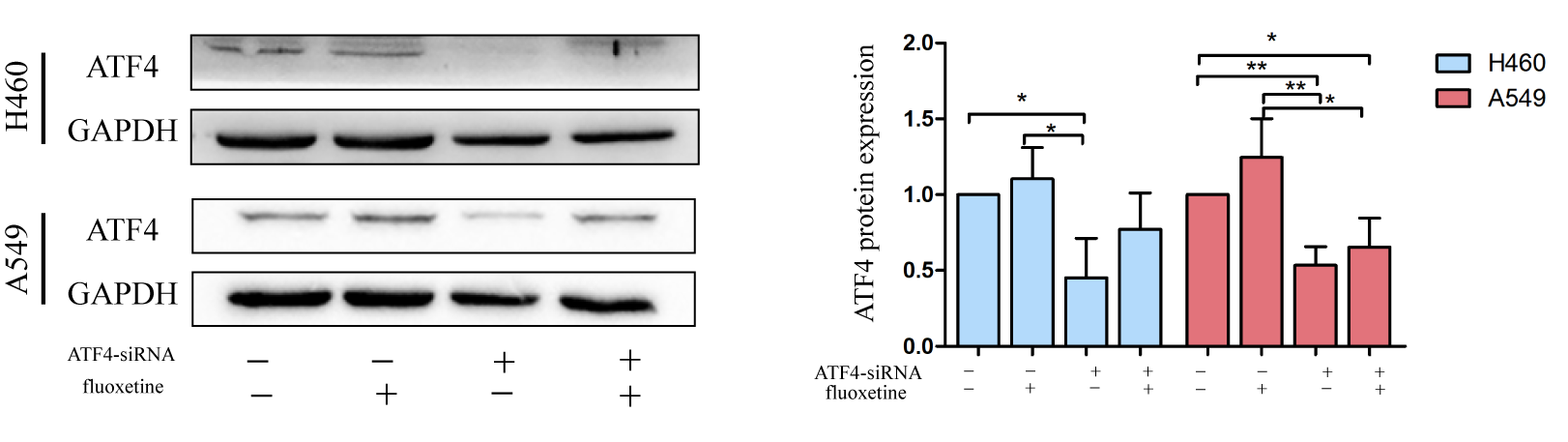

Supplement: Supplementary file 2 [file Image2.TIF]

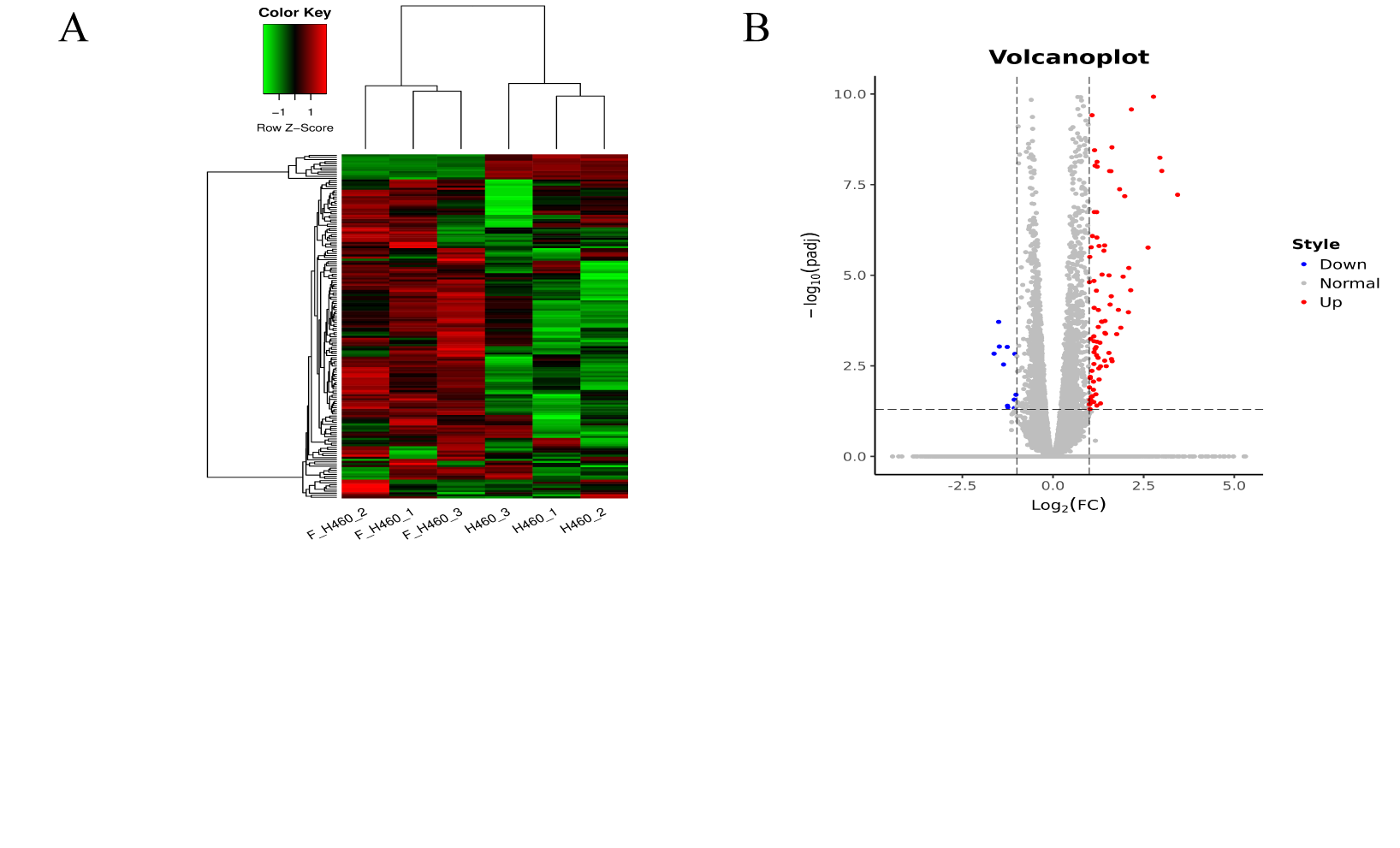

Supplement: Supplementary file 3 [file Image1.TIF]
